# Biallelic Missense Mutation in the *ECEL1* Underlies Distal Arthrogryposis Type 5 (DA5D)

**DOI:** 10.3389/fped.2019.00343

**Published:** 2019-08-28

**Authors:** Muhammad Umair, Amjad Khan, Amir Hayat, Safdar Abbas, Abdulaziz Asiri, Muhammad Younus, Wajid Amin, Shoaib Nawaz, Shazia Khan, Erum Malik, Majid Alfadhel, Farooq Ahmad

**Affiliations:** ^1^Medical Genomics Research Department, King Abdullah International Medical Research Center (KAIMRC), Riyadh, Saudi Arabia; ^2^Ministry of National Guard - Health Affairs (MNGHA), King Saud bin Abdulaziz University for Health Sciences, Riyadh, Saudi Arabia; ^3^Department of Developmental Medicine, King Abdullah International Medical Research Center (KAIMRC), Riyadh, Saudi Arabia; ^4^Department of Biochemistry, Faculty of Life and Chemical Sciences, Abdul Wali Khan University, Mardan, Pakistan; ^5^Department of Biochemistry, Faculty of Biological Sciences, Quaid-i-Azam University, Islamabad, Pakistan; ^6^State Key Laboratory of Membrane Biology, Beijing Key Laboratory of Cardiometabolic Molecular Medicine, Institute of Molecular Medicine, Peking-Tsinghua Center for Life Sciences, PKU-IDG/McGovern Institute for Brain Research, Peking University, Beijing, China; ^7^Immunology and Genomic Medicine Lab, Graduate School of Medicine, Kyoto University, Kyoto, Japan; ^8^Department of Biological Sciences, International Islamic University Islamabad, Islamabad, Pakistan; ^9^Department of Biochemistry, Shah Abdul Latif University Khairpur, Sindh, Pakistan; ^10^Division of Genetics, Department of Pediatrics, King Abdullah Specialized Children Hospital, Riyadh, Saudi Arabia; ^11^Department of Chemistry, Women University Swabi, Swabi, Pakistan

**Keywords:** distal arthrogryposis, DA5D, *ECEL1*, contractures, missense mutation

## Abstract

Distal arthrogryposis (DA) is a heterogeneous sub-group of arthrogryposis multiplex congenita (AMC), mostly characterized by having congenital contractures affecting hands, wrists, feet, and ankles. Distal arthrogryposis is mostly autosomal dominantly inherited, while only one sub-type DA type 5D is inherited in an autosomal recessive manner. Clinically, DA5D is described having knee extension contractures, micrognathia, distal joint contractures, clubfoot, ptosis, contractures (shoulders, elbows, and wrists), and scoliosis. Using whole exome sequencing (WES) followed by Sanger sequencing, we report on a first familial case of DA5D from Pakistani population having a novel biallelic missense mutation (c.158C>A, p.Pro53Leu) in the *ECEL1* gene. Our result support that homozygous mutations in *ECEL1* causes DA5D and expands the clinical and allelic spectrum of *ECEL1* related contracture syndromes.

## Introduction

Arthrogryposis multiplex congenita (AMC) is a heterogeneous disorder mainly characterized by congenital contractures of two or more than two joints. AMC incidence rate is 1/3,000 live births affecting both sexes equally (1, 2). Distal arthrogryposis (DA) is defined as the condition with non-progressive contractures and affecting the distal joints (i.e. hands, feet, wrists, and ankles) with limited proximal joints involvement. DA is caused by pathogenic mutations in the genes encoding the contractile apparatus of myofibers (2). DA overlaps clinical features with the other syndromes having both variable inter and intra-familial clinical features (1, 3, 4).

Most sub-types of DA are inherited in an autosomal dominant fashion (OMIM-listed subtypes). The genetic etiology has been determined for only six autosomal dominant and one autosomal recessive sub-type including DA1 (OMIM 108120) caused by mutation in the *TPM2* gene located on chromosome 9p13.3 (5), DA2A, DA2A7, and DA2A8 (OMIM 193700, 158300, 178110) caused by *MYH3* gene mutation located on chromosome 17p13.1 (6), DA2B (OMIM 601680) caused by heterozygous mutations in three genes *MYH3, TNNI2*, and *TNNT3* (MIM 601680) (7), DA3A and DA5A caused as a result of heterozygous mutations in the *PIEZO2* gene (OMIM 114300, 108145) located on chromosome 18p11.22-p11.21, DA9 (*FBN2*; OMIM 121050) located on chromosome 5q23.3, while gene for DA10 located on chromosome 2q31.3-q32.1 (187370) has not been identified yet (8–10). DA5D (*ECEL1*; OMIM 615065) is the only type inherited in an autosomal recessive fashion, caused by pathogenic homozygous sequence variants in the endothelin-converting enzyme-like 1 (*ECEL1*) gene (OMIM 605896), located on chromosome 2q37.1.

Herein, we describe two affected siblings with the clinical features of DA type 5D, in whom a homozygous missense variant has been identified in the *ECEL1* gene.

## Case Presentation

### Methods

#### Ethical Approval

We investigated a Pakistani family recruited from remote FR Bannu region of KPK province with distinctive distal arthrogryposis like phenotypes. The authors obtained written informed consent agreements from the affected, patients and other family members (both English and local language) for publication of this case report, photographs and radiographs in compliance with the Helsinki Declaration.

#### Blood Sampling and DNA Isolation

**Figure 1:**
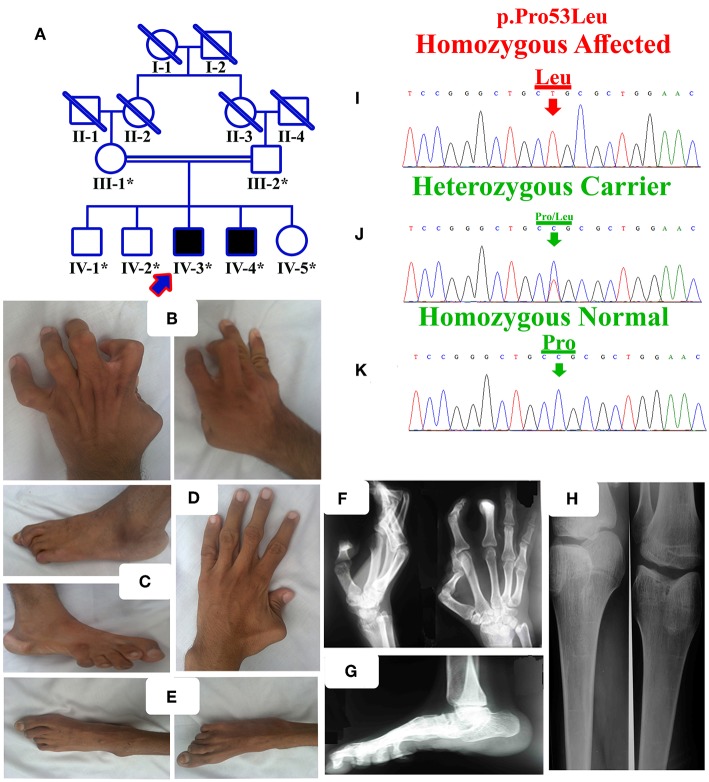
**(A)** Pedigree of the present family segregating distal arthrogryposis type 5 in autosomal recessive manner. Double lines are indicative of consanguineous unions. The individual numbers labeled with asterisks indicate the samples which were available for this study. **(B)** IV-3 having bilateral metacarpal and inter-phalangeal extension and flexion contractures. **(C)** Bilateral metatarsals extension, pes cavus, high arch, talipes varus, and overlapping toes. **(D)** IV-4 having left hand thumb flexion contracture, while normal right hand. **(E)** Bilateral metatarsals extension, pes cavus and talipes varus. **(F,G)** Radiographs of hands and feet of the affected individual IV-3, showing flexion contractures. **(H)** Both knees slightly flexed, with limited active knee flexion. **(I)** Sanger sequencing electrograms of the identified mutation, an upper panel showing the mutated nucleotide sequence (c.158C>A; p.Pro53Leu) in a homozygous state in the affected individuals, **(J)** middle panel showing heterozygous carrier and **(K)** lower panel showing the homozygous wild-type sequence.

The family was examined for all the primary and secondary features, pedigree (Figure 1A) was constructed and blood samples were collected in EDTA tubes (BD, Franklin Lakes, NJ, USA) from all the available affected and normal individuals (Figure 1A). A detailed interview with the parents/elders highlighted different prenatal, postnatal, and neonatal problems with affected individuals. Subsequently, DNA extraction and quantification was performed using standard methods.

#### Whole Exome Sequencing

DNA of the affected individual (IV-3) was subjected to whole exome sequencing (WES) using the Illumina platform following standard methods (Illumina, Inc., San Diego, Calif, USA). After exome enrichment, all the reads obtained were aligned in contradiction with the human assembly hg19 (GRCh37) using Burrows-Wheeler Aligner (BWA v 0.7.5) and variants were called using different tools such as PINDEL (v 0.2.4t), SAM tools (v 0.1.18) and Exome Depth (v1.0.0). The final VCF file (variant calling format) was analyzed using BaseSpace (Illumina; https://basespace.illumina.com/) and variant filtration was performed step-by-step using standard methods as described earlier (11, 12).

#### Sanger Sequencing

The variant identified after WES filtering were bi-directionally Sanger sequenced using standard methods (13). The complete gene sequence was retrieved from Ensembl genome browser (https://asia.ensembl.org/index.html) and primers were designed using primer3 (http://bioinfo.ut.ee/primer3/; primers sequences available on request). Sanger sequencing was performed in all the available affected and unaffected individuals of the family.

### Pathogenicity Index

The pathogenicity index for the detected variant was calculated using Mutation Taster (http://www.mutationtaster.org/), SIFT (http://sift.bii.a-star.edu.sg/), and PolyPhen-2 (http://genetics.bwh.harvard.edu/pph2/). The frequency of the variant in the general population was determined using ExAC (http://exac.broadinstitute.org/), gnomAD (http://gnomad.broadinstitute.org/), 1000 Genomes, 165 Pakistani exomes, and “Pakistan Genetic mutation database” (14).

### *In silico* Analysis

The primary sequence of ECEL1 was retrieved from Uni Prot protein database (https://www.uniprot.org/uniprot/O95672). Retrieved sequence was used to predict the 3D protein structure using I-TASSER server (15). Hydrogen atoms were added and all the water molecules were removed. Five structures were generated by server which were validated by PROCHECK, ERRAT, and VERIFY 3D. Subsequently, the refined structure were chosen for protein modeling. The three-dimensional model of mutated ECEL1 protein (p.Pro53Leu) was generated by MODELER 9.17. Structural analysis was performed using PyMOL and Chimera (1.12). Analysis of both normal and mutant ECEL1 was performed using ProSA plot, which showed Z scores between 0.5 and 1.0, indicating no significant deviation from the scores determined for proteins of similar size. The CUPSAT, SDM, and mCSM were employed to study and predict the impact of single-point mutations on protein stability.

## Results

### Clinical Evaluation

The present family has two affected individuals (IV-3 and IV-4) in the fourth generation and five unaffected individuals (III-1, III-2, IV-1, IV-2, and IV-5). Digital images for both affected individuals were obtained, while radiographs of only one affected individual (IV-3) were obtained (Figures 1B–H). Growth parameters at birth were not available. After birth, both the affected individuals had flexion deformities involving the knees, digits and elbows. The ages of affected individual examined were IV-3:16 years and IV-4:18 years. Both the affected individuals showed features such as mild scoliosis, poor weight/height gain, nasal tone speech, mild facial dysmorphism, strabismus, adducted thumbs, ptosis, camptodactyly, stiff wrists, limited knee flexion, progressive weakness of hip flexors, and pes cavus. They suffered from knees, elbows contractures, with flexion contracture of digits. All phalanges had fixed flexion deformity, although they had a firm grip (Figures 1B,D). Feet were thin, stiff, and the toes had restricted movements (Figures 1C,E).

### Radiographic Examination

The affected individual (IV-3) showed flexion deformity of digits in both hands and feet. Knees also revealed flexion contracture at the beginning, later after surgery, he was able to bend the knee and walk without support. Foot radiographic examination revealed vertical talus and fixed flexion deformity (contracture) of the toes.

Both sibs performed very well in school and underwent knee surgery, after which they were able to bend the knee and walk without support. Abnormalities such as hearing, dental anomalies, vision, and intellectual disability were not observed at the time of clinical examination. The affected individuals did not reveal any features such as polydactyly, syndactyly, split hand-foot malformation, or some other related skeletal abnormality since the family was initially examined. Detail clinical evaluation of the two affected individuals has been presented in Table 1.

**Table 1:** Clinical examination of the two affected individuals.

**S. No**.	**Features**	**IV-3**	**IV-4**
1	Origin	Pakistani	Pakistani
2	Ethnicity	Muslim	Muslim
3	Mutation	c.158C>T, p.Pro53Leu	c.158C>T, p.Pro53Leu
4	Consanguinity	+	+
5	Reduced fetal movement	–	–
6	Delivery by CS	–	–
7	Age at last examination	16	18
8	Antenatal contractures	+	+
9	Learning difficulties	–	–
10	Facial dysmorphism	+	+
11	Ptosis	+	+
12	Camptodactyly	+	+
13	Opthalmoplegia	–	–
14	Celft palate	–	–
15	Strabismus	+	+
16	Central tongue groove	–	–
17	Neck webbing	–	–
18	Micrognatha	–	–
19	Scoliosis	–	–
20	Finger contracture	+	+
21	Limitation in elbow movements	+	+
22	Limitation in knee movements	+	+
23	Ankle contractures	+	+
24	Proximal or distal weakness	–	–
25	Respiratory infection	–	–
26	Hearing disorder	–	–
27	Dentinogenesis imperfecta	–	–
28	Muscular dystrophy	+	–
29	Hearing impairment	–	–
30	Intellectual disability	–	–

### Whole Exome Sequencing

WES using DNA of the single affected individual (IV-3) was performed using standard Illumina platform, and filtering steps were performed as previously described (11, 12). The final VCF file (variant calling format) was uploaded and analyzed online using Illumina BaseSpace (Illumina; https://basespace.illumina.com/) software. As pedigree depicted autosomal recessive inheritance (Figure 1A), so first we screened the VCF file for homozygous variants, although compound heterozygous, *de novo* and heterozygous variants were not ignored. Filtering process revealed a homozygous missense mutation (c.158C>A; p.Pro53Leu) in the *ECEL1* gene (Table 2). Detail of the WES coverage metrics for the affected individual IV-3 is presented in Supplementary Table 1.

**Table 2:** Filtering steps followed to identify the disease causing variant.

**Filtration Steps**	**Number of variants detected**
Total variants detected in affected individual (IV-3)	78,507
Total heterozygous variants detected (autosomal dominant or *de novo)*	47,244
Total homozygous variants detected (autosomal recessive inheritance)	31,263
Total variants after dbsnp exclusion	3,539
Total homozygous frameshift variants detected	186
Total homozygous indels detected	89
Total homozygous missense variants detected	77
Total homozygous non-sense variants detected	2
Total homozygous splice site variants detected	41
Total homozygous near splice site variants detected	17
Total homozygous synonymous variants detected	18
Total homozygous unknown variants detected	18
Total homozygous 3′ and 5′ UTR variants detected	311
Total homozygous variants identified after applying different filters (NHLBI-ESP; 1000 Genomes; ExAC) with MAF > 0.001	32
Total compound heterozygous variants identified after applying different filters (NHLBI-ESP; 1000 Genomes; ExAC) with MAF > 0.001	5
Homozygous variant identified in known or predicted to be involved in the disease of interest	1

### Pathogenicity Index

The pathogenicity index of the identified mutation was determined using different online tools represented in Supplementary Table 2 and the mutation was classified as damaging by most of the tools.

### *ECEL1* Sanger Sequencing

Sanger sequencing of the identified mutation was performed using standard methods as described previously (13). The mutation (c.158C>A; p.Pro53Leu) segregated perfectly with the disease phenotype (Figures 1I–K).

### *In silico* Analysis

**Figure 2:**
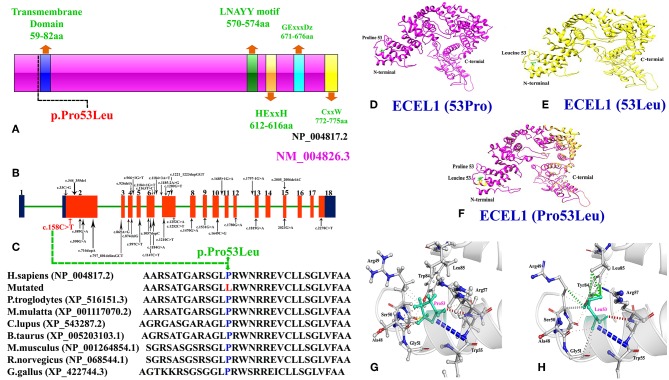
**(A)** Schematic representation of ECEL1 protein and domains including the transmembrane domain (TM: 59-82aa; exon 2), the LNAYY motif (exon 11) involved in substrate orientation, the HExxH zinc binding motif (exon 13), the GExxxD zinc coordinating motif (exon 15) and the CxxW metalloprotease conserved carboxy terminal sequence (exon 18). The mutation identified in the present case study lie next to the N-terminal transmembrane domain. **(B)** Cartoonic representation of *ECEL1* exons, black arrows represent already reported variants and red arrow represent the variant identified in the present study. Exons and introns are not drawn up to scale. **(C)** Amino acid sequence comparison of human ECEL1 protein with other orthologs species showing conservation of Pro53 residue across different species. **(D)** Wild-type ECEL1 protein structure showing Proline at amino acid position 53. **(E)** Mutant ECEL1 protein structure showing Leucine at amino acid position 53. **(F)** Representing difference in the protein structure after alignment of both mutant and wild type protein. **(G)** Close-up view of the wild type Proline 53 and **(H)** close-up view of the mutant Leucine 53 showing additional interactions.

We performed protein structure prediction for the novel homozygous missense mutation (c.158C>A, p.Pro53Leu) in the *ECEL1* gene. The residue Proline53 is located in the cytoplasmic domain and missense mutations at this position might affect the secondary structure of the protein (Figures 2D–F). Our analysis revealed that Pro53 interact with Arg49, Ala48, Ser50, Arg57, and Leu85. Substitution of a non-polar Proline to a non-polar Leucine do not introduce new H-bond interactions but Leucine due to its different structure may interact with surrounding amino acid residues differently and these new interactions in turn might potentially disrupt both protein secondary structure and function (Figures 2G,H). Using CUPSAT, SDM, and mCSM we predicted that pro53Leu mutation would cause a −0.66, −0.586, and −0.63 kcal/mole change in the ΔΔG, respectively, indicating that the mutation would greatly destabilize the protein structure and hence disrupted function.

## Discussion

Mutations in the *ECEL1* (NM_004826.3) gene have been previously reported causing distal arthrogryposis phenotypes with no clear genotype-phenotype correlations. Phenotypes such as ptosis, distinct facial features, severe hand camptodactyly, toe and foot contractures, and knee deformities reported earlier (3, 16–18) were also observed in the present affected individuals. While, Shaheen et al. (19) reported incomitant strabismus in three affected individuals and Ullmann et al. (9) reported the same feature in 1/7 affected individuals, this feature was also observed in both the affected individuals reported here. Moreover, respiratory issues reported earlier were also observed in our affected individuals (9, 19). Some of the features such as deep central groove in the tongue, digital webbing, spine issue and cleft palate reported by Ullmann et al. (9), were not observed in our patients.

Consanguineous marriages have become an alarming issue in a culturally rich and religious country like Pakistan, which increase the likelihood for single gene disorder (autosomal recessive). It has been estimated that almost 82.5% of Pakistani parents are blood relatives (20, 21). In such a situation, next generation sequencing technologies such as WES and whole genome sequencing (WGS) is a preferred choice for quick and efficient molecular diagnostic tool in single gene disorders.

Keeping this in mind, we used WES to identify the molecular cause of the disease in both the affected individuals in the family. WES followed by bi-directional Sanger sequencing identified a homozygous missense mutation (c.158C>A; p.Pro53Leu) that segregated perfectly with the disease phenotype. Homology modeling of the normal and mutant ECEL protein revealed substantial changes in the N-terminal of the ECEL1 protein, which might result in the change in the secondary structure of the mutated protein (Figures 2D–H). Structural analysis showed that the mutation (Pro53Leu) would greatly destabilize the protein structure. Amino acid sequence comparison of human ECEL1 protein with other orthologs species showed that the Pro53 residue is highly conserved across different species (Figure 2C).

To date, only 32 mutations have been identified in the *ECEL1* gene in patients from different ethical backgrounds causing arthrogryposis multiplex congenital with axoglial defects, contractual syndrome and distal arthrogryposis type 5. These mutations include 13 missense, 6-splice site, 4 non-sense, 4 small deletions, 4 small duplications, 1 regulatory region (Figures 2A,B; Supplementary Table 3). Most mutations in the *ECEL1* gene were reported in the exon 2 that corresponds to the transmembrane domain (TM; 59aa-82aa) of the ECEL1 protein. The mutation identified in our patients also lies near to the TM domain, highlighting the importance of this domain in the proper function of ECEL1 protein.

*ECEL1* encodes the endothelin-converting enzyme-like 1, a membrane-bound metalloproteinase, which is similar in structure to the endothelin-converting enzyme (ECE) (21). It is highly expressed in neurons within the peripheral and central nervous system and belongs to the neprilysin family of zinc metalloendopeptidases (2, 22–25). *Ecel1*^−/−^ mice die early due to respiratory failure after birth (22, 23). Similarly, homolog knock-in rodent animal model demonstrate a decrease in peripheral motor axons, having a decrease in final branching of the diaphragm, skeletal muscles nerve terminals, and fail to form an adequate number of neuromuscular junctions (NMJs) (22).

To the best of our knowledge, the present study is the first report *ECEL1* gene mutation causing rare DA syndrome from the Pakistani population and adds to the limited knowledge of rare DA syndrome related to contractual disorder pathogenesis. The present report further expands the *ECEL1* mutation spectrum, which might help in genotype-phenotype correlations and highlights the importance of including DA5D in the differential for multiple pterygium syndromes.

## Data Availability

The raw data supporting the conclusions of this manuscript will be made available by the authors, without undue reservation, to any qualified researcher.

## Ethics Statement

This study was carried out in accordance with Declaration of Helsinki approved by Institutional IRB committee and written informed consent was obtained from all subjects.

## Author Contributions

MU drafted the manuscript. MU, MY, WA, EM, SN, SK, AA, and AK collected the samples, clinical data, analyzed data, and performed experiments. SA and AH performed the *in silico* analysis. MU, AA, and AK analyzed the genomic data. MA and FA edited the manuscript. MU, FA, and MA conceived and designed the research study.

### Conflict of Interest Statement

The authors declare that the research was conducted in the absence of any commercial or financial relationships that could be construed as a potential conflict of interest.

## References

[1] Bamshad M Jorde LB Carey JC. Arevised and extended classification of the distal arthrogryposes. Am J Med Genet. (1996) 65:277–81. 10.1002/(SICI)1096-8628(19961111)65:4<277::AID-AJMG6>3.0.CO;2-M 8923935

[2] Dieterich K Quijano-Roy S Monnier N Zhou J Faure J Smirnow DA The neuronal endopeptidase ECEL1 is associated with a distinct form of recessive distal arthrogryposis. Hum Mol Genet. (2013) 15:1483–92. 10.1093/hmg/dds514 23236030

[3] Nagata K Kiryu-Seo S Tamada H Okuyama-Uchimura F Kiyama H Saido TC. ECEL1 mutation implicates impaired axonal arborization of motor nerves in the pathogenesis of distal arthrogryposis. Acta Neuropathol. (2016) 132:111–26. 10.1007/s00401-016-1554-0 26951213

[4] Stattin EL Johansson J Gudmundsson S Ameur A Lundberg S Bondeson ML. A novel ECEL1 mutation expands the phenotype of distal arthrogryposis multiplex congenita type 5D to include pretibial vertical skin creases. Am J Med Genet A. (2018) 176:1405–10. 10.1002/ajmg.a.38691 29663639

[5] Sung SS Brassington AM Grannatt K Rutherford A Whitby FG Krakowiak PA. Mutations in genes encoding fast-twitch contractile proteins cause distal arthrogryposis syndromes. Am J Hum Genet. (2003) 72:681–90. 10.1086/368294 12592607 PMC1180243

[6] Toydemir RM Rutherford A Whitby FG Jorde LB Carey JC Bamshad MJ. Mutations in embryonic myosin heavy chain (MYH3) cause Freeman-Sheldon syndrome and Sheldon-Hall syndrome. Nat Genet. (2006) 38:561–5. 10.1038/ng1775 16642020

[7] Sung SS Brassington AM Krakowiak PA Carey JC Jorde LB Bamshad M. Mutations in TNNT3 cause multiple congenital contractures: a second locus for distal arthrogryposis type 2B. Am J Hum Genet. (2003) 73:212–4. 10.1086/376418 12865991 PMC1180583

[8] Toydemir RM Chen H Proud VK Martin R van Bokhoven H Hamel BC. Trismus-pseudocamptodactyly syndrome is caused by recurrent mutation of MYH8. Am J Med Genet A. (2006) 140:2387–93. 10.1002/ajmg.a.31495 17041932

[9] Ullmann U D'Argenzio L Mathur S Whyte T Quinlivan R Longman C. ECEL1 gene related contractural syndrome: long-term follow-up and update on clinical and pathological aspects. Neuromuscul Disord. (2018)28:741–9. 10.1016/j.nmd.2018.05.012 30131190

[10] Rai A Puri RD Phadke SR. Extending the phenotype and an ECEL1 gene mutation in distal arthrogryposis type 5D. Clin Dysmorphol. (2018) 27:130–4. 10.1097/MCD.0000000000000236 30080694

[11] Umair M Shah K Alhaddad B Haack TB Graf E Strom TM. Exome sequencing revealed a splice site variant in the IQCE gene underlying post-axial polydactyly type A restricted to lower limb. Eur J Hum Genet. (2017) 25:960–5. 10.1038/ejhg.2017.83 28488682 PMC5567151

[12] Umair M Alhaddad B Rafique A Jan A Haack TB Graf E. Exome sequencing reveals a novel homozygous splice site variant in the WNT1 gene underlying osteogenesis imperfecta type 3. Pediatr Res. (2017) 82:753–8. 10.1038/pr.2017.149 28665926

[13] Umair M Hassan A Jan A Ahmad F Imran M Samman MI. Homozygous sequence variants in the FKBP10 gene underlie osteogenesis imperfecta in consanguineous families. J Hum Genet. (2016) 61:207–13. 10.1038/jhg.2015.129 26538303

[14] Qasim I Ahmad B Khan MA Khan N Muhammad N Basit S. Pakistan genetic mutation database (PGMD); a centralized Pakistani mutome data source. Eur J Med Genet. (2018) 61:204–8. 10.1016/j.ejmg.2017.11.015 29223505

[15] Yang J Yan R Roy A Xu D Poisson J Zhang J. The I-TASSER Suite: protein structure and function prediction. Nat Methods. (2015) 12: 7–8. 10.1038/nmeth.3213 25549265 PMC4428668

[16] Patil SJ Rai GK Bhat V Ramesh VA Nagarajaram HA Matalia J. Distal arthrogryposis type 5D with a novel ECEL1 gene mutation. Am J Med Genet A. (2014) 164A:2857–62. 10.1002/ajmg.a.36702 25099528

[17] Dohrn N Le VQ Petersen A Skovbo P Pedersen IS Ernst A. ECEL1 mutation causes fetal arthrogryposis multiplex congenita. Am J Med Genet A. (2015) 167A:731–43. 10.1002/ajmg.a.37018 25708584

[18] Hamzeh AR Nair P Mohamed M Saif F Tawfiq N Khalifa M. A novel variant in the endothelin-converting enzyme-like 1 (ECEL1) gene in an emirati child. Med Princ Pract. (2017) 26:195–8. 10.1159/000456034 28114145 PMC5588382

[19] Shaheen R Al-Owain M Khan AO Zaki MS Hossni HA Al-Tassan R. Identification of three novel ECEL1 mutations in three families with distal arthrogryposis type 5D. Clin Genet. (2014) 85:568–72. 10.1111/cge.12226 23829171

[20] Umair M Ahmad F Ullah A Whole exome sequencing as a diagnostic tool for genetic disorders in Pakistan. Pak J Med Res. (2018) 57:97–8.

[21] Umair M Ahmad F Bilal M Asiri A Younus M Khan A A comprehensive review of genetic skeletal disorders reported from Pakistan: a brief commentary. Meta Gene. (2019) 20:100559 10.1016/j.mgene.2019.100559

[22] Nagata K Kiryu-Seo S Maeda M Yoshida K Morita T Kiyama H. Damage-induced neuronal endopeptidase is critical for presynaptic formation of neuromuscular junctions. J Neurosci. (2010) 30:6954–62. 10.1523/JNEUROSCI.4521-09.2010 20484637 PMC6632665

[23] Shaaban S Duzcan F Yildirim C Chan WM Andrews C Akarsu NA. Expanding the phenotypic spectrum of ECEL1-related congenital contracture syndromes. Clin Genet. (2014) 85:562–7. 10.1111/cge.12224 23808592 PMC3883930

[24] Schweizer A1 Valdenaire O Köster A Lang Y Schmitt G Lenz B. Neonatal lethality in mice deficient in XCE, a novel member of the endothelin-converting enzyme and neutral endopeptidase family. J Biol Chem. (1999) 274:20450–6. 10.1074/jbc.274.29.20450 10400672

[25] Shirotani K Tsubuki S Iwata N Takaki Y Harigaya W Maruyama K. Neprilysin degrades both amyloid beta peptides 1–40 and 1–42 most rapidly and efficiently amongthiorphan- and phosphoramidon-sensitive endopeptidases. Biol Chem. (2001) 276:21895–901. 10.1074/jbc.M008511200 11278416

