# Supplementary material for: Biallelic Missense Mutation in the ECEL1 Underlies Distal Arthrogryposis Type 5 (DA5D)
Source: Front Pediatr. 2019 Aug 28;7:343. doi: 10.3389/fped.2019.00343 (PMC6724761; doi:10.3389/fped.2019.00343)
Supplement: Supplementary file 1 [file Table_1.DOCX]

**Supplementary table 1:** Detail of the coverage metrics for the affected individual IV-3.

| **Sample** | IV-3 |
| --- | --- |
| **Sex** | Male |
| **Pedigree** | Autosomal Recessive |
| **Reads** | 108449843 |
| **Percent** | 98.87 |
| **Avg Cov** | 134.54 |
| **Uncovered** | 0.11 |
| **Cov 1x** | 99.89 |
| **Cov 4x** | 99.75 |
| **Cov 8x** | 99.6 |
| **Cov 20x** | 98.34 |
| **Cov 40x** | 98.22 |
