# Supplementary material for: Biallelic Missense Mutation in the ECEL1 Underlies Distal Arthrogryposis Type 5 (DA5D)
Source: Front Pediatr. 2019 Aug 28;7:343. doi: 10.3389/fped.2019.00343 (PMC6724761; doi:10.3389/fped.2019.00343)
Supplement: Supplementary file 2 [file Table_2.DOCX]

**Supplementary Table 2:** Pathogenicity of the identified variant (c.158C>T, p.Pro53Leu) using different online available tools.

| **S.No** | **Bioinformatics tool** | **Results** | **Score** |
| --- | --- | --- | --- |
| 1 | **MutationTaster** | Disease causing | **1** |
| 2 | **DANN** | Pathogenic | **0.9897** |
| 3 | **SIFT** | Damaging | **0.005** |
| 4 | **FATHMM** | Damaging | **-1.5** |
| 5 | **Varsome** | Uncertain Significance | **PM2 (pathogenic)** |
| 6 | **FATHMM-MKL** | Damaging | **0.9303** |
| 7 | **MetaSVM** | Tolerated | **-0.2842** |
| 8 | **Provean** | Neutral | **-2.26** |
