# Supplementary material for: Biallelic Missense Mutation in the ECEL1 Underlies Distal Arthrogryposis Type 5 (DA5D)
Source: Front Pediatr. 2019 Aug 28;7:343. doi: 10.3389/fped.2019.00343 (PMC6724761; doi:10.3389/fped.2019.00343)
Supplement: Supplementary file 3 [file Table_3.DOCX]

**Supplementary Table 3:** Mutations reported to-date in the *ECEL1* gene.

| **Mutation Type** | **Nucleotide change** | **Amino**  **acid change** | **Phenotype** | **Exon/ Intron** | **Population ExAC Frequency** | **Reported population** |
| --- | --- | --- | --- | --- | --- | --- |
| **Missense** | c.590G>A | p.Gly197Asp | Arthrogryposis, distal, type 5D | Exon 2 | 0.0000319 | USA |
| **Missense** | c.589G>A | p.Gly197Ser | Contractural syndrome | Exon 2 | 0.00000443 | UK |
| **Missense** | c.869A>G | p.Try290Cys | Arthrogryposis, distal, type 5D | Exon 4 | 0.00 | USA |
| **Missense** | c.1163T>C | p.Leu388Pro | Arthrogryposis, distal, type 5D | Exon 6 | 0.00 | Sweden |
| **Missense** | c.1184G>A | p.Arg395Gln | Arthrogryposis, distal, type 5D | Exon 6 | 0.00 | USA |
| **Missense** | c.1209G>T | p.Trp403Cys | Arthrogryposis multiplex congenita | Exon 7 | 0.00 | Qatar |
| **Missense** | c.1210C>T | p.Arg404Cys | Arthrogryposis, distal, type 5D | Exon 7 | 0.0000121 | Turkish |
| **Missense** | c.1252C>T | p.Arg418Cys | Arthrogryposis, distal, type 5D | Exon 7 | 0.00000799 | USA |
| **Missense** | c.1531G>A | p.Gly511Ser | Arthrogryposis, distal, type 5D | Exon 9 | 0.0000042 | Australia. |
| **Missense** | c.1780G>A | p.Asp594Asn | Developmental disorder | Exon 12 | 0.0000159 | Deciphering Developmental Disorders Study |
| **Missense** | c.1819G>A | p.Gly607Ser | Arthrogryposis multiplex congenita with ophthalmplegia | Exon 13 | 0.00 | USA and Japan |
| **Missense** | c.2023G>A | p.Ala675Thr | Arthrogryposis, distal, type 5D | Exon 15 | 0.000011 | India, Denmark and Turkish |
| **Missense** | c.2278T>C | p.Cys760Arg | Arthrogryposis, distal | Exon 18 | 0.00 | Japan |
| **Nonsense** | c.997C>T | p.Arg333* | Arthrogryposis, distal | Exon 5 | 0.0000279 | France |
| **Nonsense** | c.1147C>T | p.Gln383* | Arthrogryposis | Exon 6 | 0.00 | Arab and Turkish |
| **Nonsense** | 1470G>A | p.Trp490* | Arthrogryposis, distal, type 5D | Exon 8 | 0.00 | USA |
| **Nonsense** | c.33C>G | p.Tyr11* | Arthrogryposis multiplex congenita with axoglial defects | Exon 2 | 0.00 | France |
| **Nonsense** | c.1649C>G | p.Ser550* | Arthrogryposis, distal | Exon 10 | 0.00 | France |
| **Splice site** | c.966+1G>A | p.? | Arthrogryposis, distal | Intron 4 | 0.00 | France |
| **Splice site** | c.1185-2A>G | p.? | Abnormality of the musculoskeletal system | Intron 6 | 0.00 | USA |
| **Splice site** | c.1184+1G>T | p.? | Arthrogryposis, distal, type 5D | Intron 6 | 0.00 | Dubai, United Arab Emirates |
| **Splice site** | c.1184+3A>T | p.? | Arthrogryposis, distal, type 5D | Intron 6 | 0.00 | USA |
| **Splice site** | c.1797-1G>A | p.? | Arthrogryposis, distal, type 5D | Intron 12 | 0.00 | Australia |
| **Splice site** | c.1685+1G>A | p.? | Arthrogryposis, distal, type 5D | Intron 10 | 0.00000398 | France |
| **Small deletions** | c.344_355del12 | p.(Asn115_Ala118del) | Arthrogryposis, distal, type 5D | Exon 2 | 0.00 | USA |
| **Small deletions** | c.874delG | p.(Val292Cysfs*51) | Arthrogryposis, distal | Exon 4 | 0.00 | France |
| **Small deletions** | c.926delA | p.(Lys309Argfs*34) | Arthrogryposis multiplex congenita with axoglial defects | Exon 4 | 0.00 | France |
| **Small deletions** | c.2005_2006delAC | p.(Thr669Alafs*39) | Arthrogryposis, distal, type 5D | Exon 15 | 0.00 | UK |
| **Small insertions** | c.716dupA | p.? | Arthrogryposis, distal, type 5D | Exon 2 | 0.00 | USA |
| **Small insertions** | c.1057dupC | p.? | Arthrogryposis, distal, type 5D | Exon 5 | 0.00 | Saudi Arabia |
| **Small insertions** | c.1221_1223dupGGT | p.? | Arthrogryposis, distal, type 5D | Exon 7 | 0.00 | Saudi Arabia |
| **Small insertions** | c.797_801delATGGGinsGCT | p.? | Arthrogryposis, distal, type 5D | Exon 3 | 0.00 | USA |
